# Aortic valve replacement in patients with significant aortic regurgitation: Comparing bicuspid and tricuspid aortic valve outcomes

**DOI:** 10.1016/j.ahjo.2025.100617

**Published:** 2025-09-12

**Authors:** Ching-Yan Zhu, Jing-Nan Zhang, Ferit Böyük, Yi-Kei Tse, Yan Chen, Mei-Zhen Wu, Qing-Wen Ren, Jia-Yi Huang, Ran Guo, Wen-Li Gu, Daniel Tai-Leung Chan, Frank A. Flachskampf, Kai-Hang Yiu

**Affiliations:** a Division of Cardiology, Department of Medicine, The University of Hong Kong-Shenzhen Hospital, Shenzhen, China; b Division of Cardiology, Department of Medicine, the University of Hong Kong, Queen Mary Hospital, Hong Kong; c Department of Medical Sciences, Cardiology and Clinical Physiology, Uppsala University and Uppsala University Hospital, Uppsala, Sweden; d Department of Cardiology, Health Science University Kanuni Sultan Süleyman Training and Research Hospital, İstanbul, Turkey; e Division of Cardiothoracic Surgery, Department of Surgery, the University of Hong Kong, Queen Mary Hospital, Hong Kong

**Keywords:** Aortic valve replacement, Aortic regurgitation, Bicuspid aortic valve, Aortic valve surgery

## Abstract

**Background:**

The role of bicuspid aortic valve (BAV) morphology in left ventricular (LV) remodelling and outcomes in aortic regurgitation (AR) patients undergoing surgical aortic valve replacement (SAVR) remains unclear. This study compares LV function and postoperative outcomes in patients with moderate-to-severe AR, stratified by BAV versus tricuspid aortic valve (TAV) morphology.

**Methods:**

We retrospectively analyzed 323 patients with isolated moderate-to-severe AR undergoing SAVR (BAV: *n* = 70; TAV: *n* = 253). Baseline clinical, echocardiographic, surgical, and follow-up data were evaluated. Adverse events were defined as a composite of all-cause mortality and heart failure rehospitalization.

**Results:**

Patients with BAV were a decade younger, were less symptomatic, but had smaller indexed aortic valve areas (AVAi) and greater regurgitant volumes. Patients with BAV had greater rise in indexed stroke volumes (SVi) with rising indexed LV end-diastolic volumes (LVEDVi) (p_interaction_ = 0.008), and milder LV ejection fraction decline with increasing LV end-systolic diameter (LVESD) (p_interaction_ = 0.004). Patients with BAV experienced less adverse events (16 % vs 30 %, *p* = 0.014). AVAi and indexed LV mass (LVMi) independently predicted adverse events after multivariable adjustment and age-matching, while valve morphology did not.

**Conclusion:**

In patients requiring SAVR for isolated AR, patients with BAV had more severe valve dysfunction at a younger age but more preserved systolic function with LV dilation. AVAi and LVMi independently predict postoperative outcomes, supporting their use for risk stratification and timely intervention, especially in younger BAV patients.

## Highlights

• Patients with BAV-AR were younger and had more advanced valvular disease before surgery.
• Patients with BAV-AR had more preserved LV function despite more severe AR.
• Indexed aortic valve area and LV hypertrophy may guide optimal AVR timing in AR.

## Graphical abstract

**Figure:**
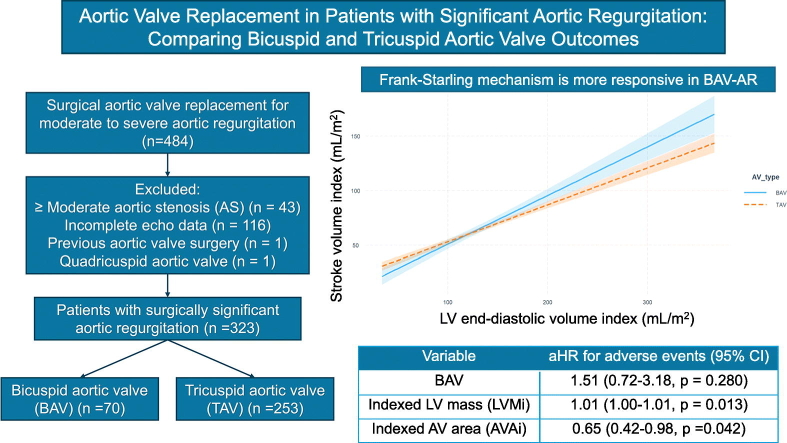

## 1 Introduction

Bicuspid aortic valve (BAV) is the most common congenital valvular heart disease, present in 1–2 % of the population [1]. Although aortic stenosis (AS) is the most common comorbidity associated with BAV, moderate or severe aortic regurgitation (AR) is also present in 13–30 % of patients [2]. In patients with both BAV or tricuspid aortic valves (TAV), timely surgical aortic valve replacement (SAVR) for AR is required [3] to definitively prevent further left ventricular (LV) ejection fraction (LVEF) decline and poor outcomes [4,5].

Previous studies demonstrate dissimilar etiologies and valvular characteristics between BAV-AR and TAV-AR, including younger age and mixed pathologies [[6], [7], [8], [9]]. However, the importance of these differences regarding LV function and postoperative adverse events remains unknown. As such, our study aims to compare valvular and ventricular characteristics in patients undergoing SAVR for BAV-AR or TAV-AR, and to evaluate predictors of postoperative outcomes to inform clinical decision-making.

## 2 Methods

### 2.1 Study population

Between January 2010 and December 2022, 484 consecutive patients undergoing SAVR at Queen Mary Hospital (QMH) for moderate and severe AR were retrospectively evaluated. Exclusion criteria were: moderate-or-greater AS (*n* = 43), incomplete echocardiographic data (*n* = 116), quadricuspid AV (n = 1), or previous AV procedures (n = 1). 323 patients (BAV: *n* = 70; TAV: *n* = 253) were included in the final analysis. The study was approved by the ethics committee of the Hospital Authority (Hong Kong West Cluster).

### 2.2 Clinical and laboratory parameters

Clinical and demographic information was collected at pre-operative echocardiography, including history of hypertension, diabetes, and dyslipidaemia, New York Heart Association (NYHA) functional class, laboratory and prescription data. Surgical mortality risk was assessed with the new European System for Cardiac Operative Risk Evaluation (EuroSCORE II).

### 2.3 Echocardiographic parameters

Preoperative 2D transthoracic echocardiography studies were performed by experienced sonographers with available systems (Vivid E9, General Electric Vingmed Ultrasound; iE33, Philips Medical Systems). Global longitudinal strain (GLS) was calculated offline using semi-automatic software (Enterprise Imaging, Agfa Healthcare).

Echocardiographic modalities include two-dimensional, M-mode, color flow, continuous-wave and pulsed-wave Doppler echocardiography. All raw images were re-examined and re-measured de novo, and suboptimal images were excluded. Measurements were indexed to body surface area when appropriate. AV morphology and BAV phenotype were determined using the Sievers and Schmidtke classification by two echocardiographers (CYZ, JNZ) [10], and were verified by direct visualization and documentation during surgery. AR severity was classified according to current recommendations [11]. Maximal and mean aortic transvalvular velocities and gradients were obtained using continuous-wave Doppler measurements with a modified Bernoulli eq. AV area (AVA) was obtained using the continuity eq. AR severity was evaluated using a multiparametric approach, including widths of the vena contracta and regurgitant jet, regurgitant volume and fraction, and presence of holodiastolic flow reversal in the descending aorta, as necessary. LV ejection fraction (LVEF), LV end-diastolic (LVEDV) and end-systolic volumes (LVESV) were obtained using the Simpson biplane method from apical four- and two-chamber views. LV mass was calculated using the Devereux formula [12]. GLS was measured using speckle-tracking echocardiography from apical four-, three-, and two-chamber views, according to current ASE recommendations [13]. Left atrial volume was measured at end-systole using Simpson's method. Pulse-wave Doppler was used to measure the transmitral flow pattern, including velocities and ratio of E and A waves.

### 2.4 Outcomes

Postoperative adverse outcomes were defined as the composite of all-cause mortality and heart failure hospitalizations (HFH), defined as the first re-admission after surgery for signs or symptoms of heart failure as diagnosed by the physician. Censoring was applied at the time of HFH or death, whichever was earlier. Postoperative events were retrieved from an electronic patient management system, with complete follow-up for all patients.

### 2.5 Statistical analysis

Continuous data are expressed as mean ± SD, and categorical variables are shown in numbers and percentages. Independent samples *t*-test was used to compare continuous variables between AV morphologies. For categorical variables, the χ^2^ test or Fisher's exact test was used. An ordinary least squares linear regression model was used to investigate the interaction between systolic function and LV parameters. Kaplan-Meier analyses with log-rank tests were used to compare adverse events, including all-cause mortality and HFH. Univariable Cox proportional hazard regression analysis was performed, followed by multivariable Cox proportional hazard regression analysis adjusting for age, sex, diabetes, hypertension, LVEF, LVESD, and EuroSCORE II. Nested Cox regression analysis was performed to compare incremental prognostic values provided by indexed AV area (AVAi) and indexed LV mass (LVMi) to conventional risk prediction models in AR including LVEF and LVESD. Sensitivity and subgroup analyses were performed to compare interactions and outcomes in age-matched patients with BAV-AR or TAV-AR. To correct for the potentially elevated flow velocity from increased preload in AR, subgroup analysis was performed including patients with LVOT VTI below 22 cm, as well as patients with LVOT diameter smaller or equal to 20 mm or over 20 mm. All statistical analyses were performed using R version 4.2.1 and SPSS for Windows (version 28.0). A two-tailed *P* value <0.05 denoted statistical significance.

## 3 Results

### 3.1 Baseline characteristics

Patients with BAV-AR were a decade younger [mean age (years) (SD): 50 (13)] than those with TAV-AR [mean age (years) (SD): 60 (13)] (Table 1). They also had less heart failure and atrial fibrillation, lower creatinine, and higher body surface areas.

**Table 1:** Baseline characteristics.

Variable	Overall	BAV, *N* = 70	TAV, *N* = 253	*p*-value
**Age, y**	**58 (14)**	**50 (13)**	**60 (13)**	**<0.001**
Male sex	249 (77 %)	57 (81 %)	192 (76 %)	0.329
Body mass index, kg/m^2^	23.7 (3.8)	24.1 (3.5)	23.6 (3.9)	0.265
**Body surface area, m**^**2**^	**1.72 (0.22)**	**1.78 (0.22)**	**1.70 (0.21)**	**0.011**
Systolic blood pressure, mmHg	133 (20)	133 (23)	133 (19)	0.881
Diastolic blood pressure, mmHg	72 (14)	73 (13)	72 (14)	0.490
Diabetes	19 (5.9 %)	1 (1.4 %)	18 (7.1 %)	0.087
Hypertension	76 (24 %)	13 (19 %)	63 (25 %)	0.269
Dyslipidaemia	40 (12 %)	10 (14 %)	30 (12 %)	0.585
Aortic aneurysm	97 (30 %)	23 (33 %)	74 (29 %)	0.560
**Heart failure**	**165 (51** **%)**	**28 (40** **%)**	**137 (54** **%)**	**0.036**
Coronary artery disease	16 (5.0 %)	2 (2.9 %)	14 (5.5 %)	0.537
**Atrial fibrillation**	**52 (16** **%)**	**4 (5.7** **%)**	**48 (19** **%)**	**0.008**
**Creatinine, mmol/L**	**110 (80)**	**91 (32)**	**115 (88)**	**0.002**
Haemoglobin, g/dL	12.94 (1.92)	13.21 (1.76)	12.87 (1.96)	0.240
Medications				
Aspirin	52 (21 %)	3 (5.6 %)	27 (14 %)	0.106
**Statins**	**88 (36** **%)**	**12 (23** **%)**	**76 (40** **%)**	**0.021**
Anticoagulation	16 (6.4 %)	2 (3.8 %)	14 (7.1 %)	0.535
RAAS inhibitors	292 (91 %)	59 (86 %)	233 (92 %)	0.095
Beta-blockers	278 (86 %)	57 (83 %)	221 (87 %)	0.309
Calcium channel blockers	203 (63 %)	38 (55 %)	165 (65 %)	0.122
**Diuretics**	**276 (86** **%)**	**53 (77** **%)**	**223 (88** **%)**	**0.017**
NYHA functional class				0.182
1	78 (24 %)	18 (26 %)	60 (24 %)	
2	174 (54 %)	43 (61 %)	131 (52 %)	
3	65 (20 %)	8 (11 %)	57 (23 %)	
4	6 (1.9 %)	1 (1.4 %)	5 (2.0 %)	
BAV type				
0		5 (7.2 %)		
1 (LN)		4 (5.8 %)		
1 (LR)		49 (71 %)		
1 (RN)		9 (13 %)		
2		2 (2.9 %)		
AR grade				0.811
Moderate	75 (23 %)	17 (24 %)	58 (23 %)	
Severe	248 (77 %)	53 (76 %)	195 (77 %)	

AR, aortic regurgitation; BAV, bicuspid aortic valve; NYHA, New York Heart Association; RAAS, renin-angiotensin-aldosterone system.

Statistically significant findings are bolded.

### 3.2 Echocardiographic and surgical characteristics

Patients with BAV-AR had smaller indexed aortic valve areas (AVAi), greater regurgitant volumes, larger ascending aortas, and lower indexed left atrial volumes (LAVi) (Table 2). They also had shorter total cross-clamp times and lower surgical risk, validated by three different surgical risk prediction algorithms.

**Table 2:** Echocardiographic and surgical characteristics.

Variable	Overall, *N* = 323	BAV, N = 70	TAV, N = 253	p-value
LV ejection fraction, %	51 (11)	50 (11)	51 (11)	0.908
LV end-diastolic volume index, mL/m^2^	131 (54)	131 (53)	132 (55)	0.878
LV end-systolic volume index, mL/m^2^	68 (38)	66 (33)	68 (39)	0.707
Stroke volume index, mL/m^2^	64 (25)	64 (28)	64 (24)	0.855
Interventricular septum thickness, mm	12.61 (2.37)	12.79 (2.16)	12.57 (2.42)	0.463
LV end-diastolic diameter index, mm/m^2^	34.6 (5.7)	34.1 (5.7)	34.8 (5.7)	0.432
LV end-systolic diameter index, mm/m^2^	25.7 (6.1)	25.1 (6.2)	25.8 (6.0)	0.366
LV posterior wall thickness, mm	11.68 (2.07)	12.03 (2.18)	11.58 (2.03)	0.126
LV mass index, g/m^2^	186 (58)	195 (63)	183 (57)	0.158
Relative wall thickness, %	41 (10)	42 (11)	40 (9)	0.315
LV global longitudinal strain (*n* = 216), %	−16.8 (4.7)	−17.7 (4.6)	−16.5 (4.7)	0.093
**AV maximal velocity, m/s**	**2.12 (0.7)**	**2.59 (0.90)**	**1.99 (0.58)**	**<0.001**
**AV maximal gradient, mmHg**	**20 (15)**	**30 (21)**	**17 (11)**	**<0.001**
**AV mean gradient, mmHg**	**10 (8)**	**16 (12)**	**9 (6)**	**<0.001**
**AV area index, cm**^**2**^**/m**^**2**^	**1.71 (0.71)**	**1.50 (0.84)**	**1.77 (0.66)**	**0.018**
AR vena contracta width, mm	6.60 (2.63)	6.89 (3.11)	6.51 (2.49)	0.366
**AR jet width, mm**	**20 (7)**	**18 (7)**	**20 (7)**	**0.016**
AR pressure half time, ms	364 (182)	360 (175)	365 (184)	0.840
**AR regurgitant volume, ml**	**67 (29)**	**75 (34)**	**65 (27)**	**0.029**
AR regurgitant fraction, %	61 (18 %)	60 (21)	61 (17)	0.663
Aortic sinus diameter index, mm/m^2^	23.7 (5.3)	22.8 (4.7)	23.9 (5.5)	0.088
ST junction diameter index, mm/m^2^	20.2 (5.3)	19.8 (4.7)	20.3 (5.4)	0.400
Ascending aorta diameter index, mm/m^2^	23.0 (5.5)	23.5 (5.4)	22.9 (5.6)	0.441
**Left atrial volume index, ml/m**^**2**^	**59 (41)**	**46 (19)**	**63 (44)**	**<0.001**
E wave, m/s	0.91 (0.37)	0.88 (0.31)	0.91 (0.38)	0.506
A wave, m/s	0.69 (0.28)	0.69 (0.28)	0.69 (0.27)	0.965
E/A ratio	1.46 (0.78)	1.41 (0.67)	1.47 (0.80)	0.589
Tricuspid regurgitation maximal velocity, m/s	2.76 (0.48)	2.73 (0.41)	2.76 (0.49)	0.753
Total bypass time (mins)	131.38 (52.80)	119.38 (44.65)	134.80 (54.55)	0.056
**Total cross-clamp time (mins)**	**109.66 (45.81)**	**98.29 (38.33)**	**112.92 (47.35)**	**0.036**
Inotropic support	138 (43 %)	25 (36 %)	113 (45 %)	0.180
Concomitant aortic surgery	41 (13 %)	9 (13 %)	32 (13 %)	0.963
**Logistic EuroSCORE**	**9.71 (11.26)**	**5.51 (5.46)**	**10.85 (12.14)**	**<0.001**
**EuroSCORE II**	**4.59 (7.54)**	**2.30 (2.54)**	**5.21 (8.30)**	**<0.001**
**SCTS logistic EuroSCORE**	**5.21 (7.58)**	**2.43 (2.55)**	**5.97 (8.29)**	**<0.001**
Bioprosthetic valve implant	141 (56 %)	29 (54 %)	112 (56 %)	0.735

LV, left ventricular; AR, aortic regurgitation.

Statistically significant findings are bolded.

### 3.3 Left ventricular remodelling and systolic function

**Fig. 1:**
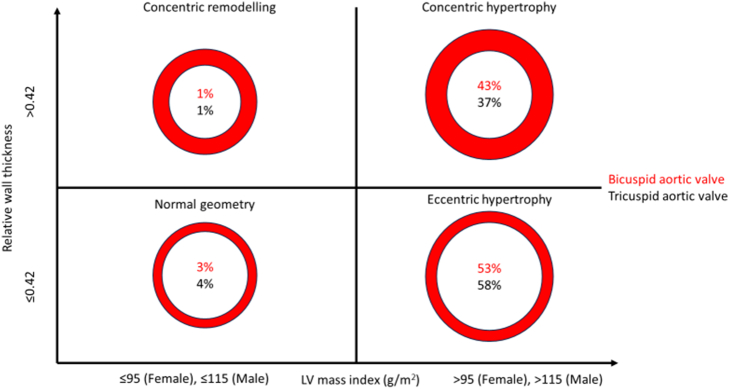
Differences in LV remodelling patterns across AV morphologies.

**Fig. 2:**
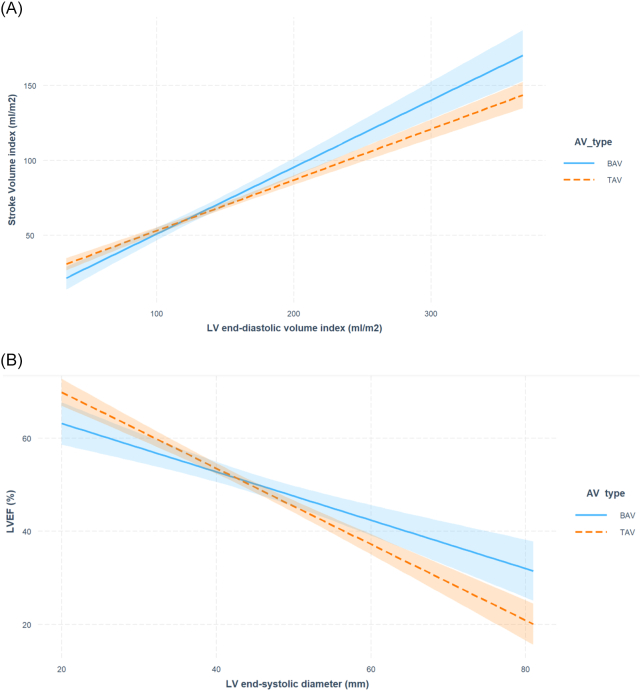
Panel A: Interaction between AV morphology and indexed LV end-diastolic volume on indexed stroke volume, with 95 % confidence intervals. P_interaction_ = 0.008; Panel B: Interaction between AV morphology and LV end-systolic diameter on left ventricular ejection fraction (LVEF), with 95 % confidence intervals. P_interaction_ = 0.004.

LV remodelling patterns across AV morphologies are shown in Fig. 1. Indexed stroke volume (SVi) rose more sharply with indexed LVEDV (LVEDVi) in patients with BAV-AR (Fig. 2, Panel A) (β = −0.720, *p* < 0.001; P_interaction_ = 0.008). Patients with BAV-AR also had a milder decline in LVEF with LVESD elevation (Fig. 2, Panel B) (β = −0.642, p < 0.001; P_interaction_ = 0.004). Both these interactions remained significant after age-matching (Supplementary fig. S2).

### 3.4 Outcomes

**Fig. 3:**
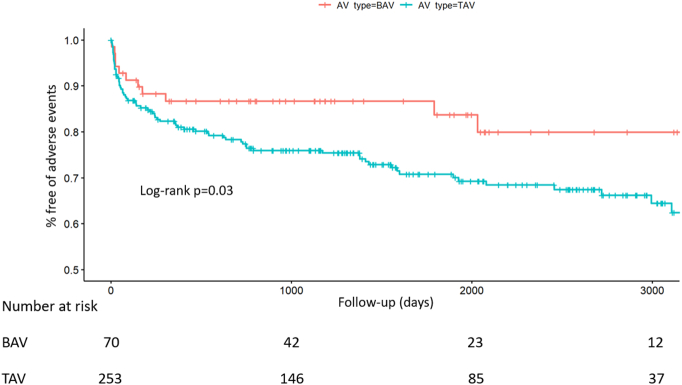
Kaplan-Meier analysis of adverse events after SAVR according to AV morphology.

Mean postoperative follow-up was similar across AV morphologies (1560 vs 1523 days, *p* = 0.826). Adverse events occurred in 88 patients (27.2 %). Patients with BAV-AR experienced less adverse events (BAV: *n* = 11 (16 %); TAV: *n* = 77 (30 %), *p* = 0.014) (Fig. 3; supplementary table 1 and supplementary fig. S1).

**Fig. 4:**
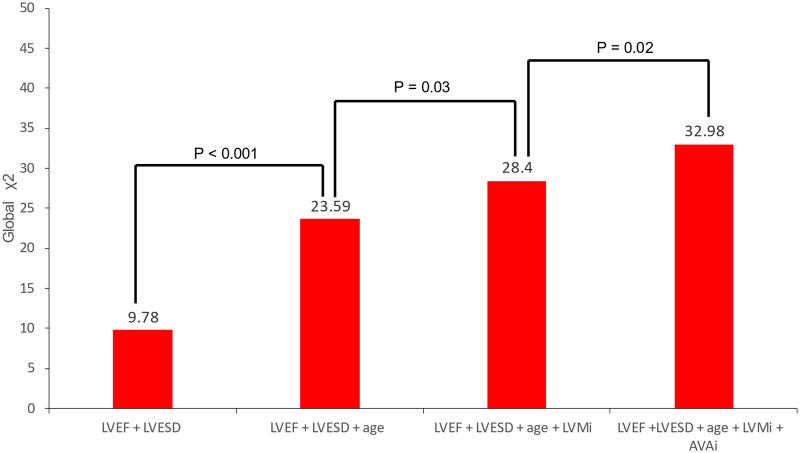
Incremental prognostic value of LVMi and AVAi when added to known variables predicting adverse events.

After multivariable Cox regression analysis, adjusting for age, sex, diabetes, hypertension, LVEF, LVESD, concomitant aortic surgery, and EuroSCORE II, AVAi (aHR per cm^2^/m^2^: 0.64 (95 % CI: 0.42–0.98, *p* = 0.039)) and LVMi (aHR per g/m^2^: 1.01 (95 % CI: 1.00–1.01, *p* = 0.013)) independently predicted adverse events, but not AV morphology (aHR for BAV: 1.51 (0.72–3.18), *p* = 0.280) (Table 3). There was no significant difference in postoperative outcomes between AVR done before 2015 or from 2015 onwards, and postoperative outcomes between BAV and TAV were similar regardless of year of AVR. The prognostic value of LVMi and AVAi was further demonstrated in a nested Cox regression analysis (Fig. 4), where prognostic models including age, LVEF and LVESD showed significantly higher prognostic value with the addition of LVMi and AVAi.

**Table 3:** Multivariable Cox regression analysis for adverse events.^a^

Variable	HR (95 % CI)	p-value
Bicuspid aortic valve	1.51 (0.72–3.18)	0.280
**Indexed LV mass (LVMi)**	**1.01 (1.00–1.01)**	**0.013**
**Indexed aortic valve area (AVAi)**	**0.64 (0.42–0.98)**	**0.039**
**Indexed left atrial volume (LAVi)**	**1.004 (1.00–1.01)**	**0.037**
Early AVR (before 2015)	0.81 (0.50–1.30)	0.374
Bicuspid aortic valve (early AVR)^b^	0.35 (0.10–1.23)	0.104
Bicuspid aortic valve (late AVR)^b^	1.10 (0.48–2.50)	0.819

Statistically significant findings are bolded.

a Adjusted for age, sex, diabetes, hypertension, LVEF, LVESD, concomitant aortic surgery, EuroSCORE II.

b Adjusted for age, diabetes, hypertension, LVEF, LVESD.

### 3.5 Sensitivity and subgroup analysis

Exact age-matching was performed on 210 patients (BAV: *n* = 60; TAV: *n* = 150) (Supplementary tables 2 and 3). AVAi and LVMi remained independently associated with adverse events (Supplementary table 4). Adverse events were similar between patients with BAV and TAV after exact age-matching (Supplementary fig. S3). The indications for concomitant aortic surgery are listed in Supplementary table 5. In subgroups including patients with LVOT VTI below 22 cm, as well as patients with LVOT diameter smaller or equal to 20 mm or over 20 mm, patients with BAV had significantly smaller AVAi than those with TAV (Supplementary Table table 6).

## 4 Discussion

This study investigated baseline, surgical, and imaging characteristics of patients requiring SAVR for isolated significant AR without significant AS, with BAV or TAV. The principal findings are: 1) patients with BAV were significantly younger and less symptomatic but had worse valvular dysfunction at the time of SAVR; 2) patients with BAV had more preserved LV systolic function with LV dilation; 3) AVAi, reflecting the valvular burden of pressure overload, was a key predictor of postoperative outcomes.

Patients with BAV-AR had more severe valvular dysfunction despite younger age and milder symptoms, highlighting the prognostic importance of BAV. Previous community-based cohorts not only demonstrate a greater prevalence of significant AR among patients with BAV, but also report concomitant AS in 25–26 % of patients with BAV-AR [14,15], compared to 17.9 % in the overall AR population [16]. BAV is also linked with higher AV velocities and more severe AR [7]. Similarly, despite younger age and better symptomatic status, patients with BAV-AR in this study had greater regurgitant volumes and smaller AVAi, possibly from cusp prolapse, sinotubular dilation [7], and heavier valvular calcification [17]. These findings highlight the necessity to understand disparities in LV remodelling and postoperative outcomes in patients with BAV-AR and TAV-AR.

Although BAV-AR presented with more severe AR, smaller AVAi in such patients may account for disparate LV remodelling and preload feedback. In patients with TAV-AR, AR confers volume overload, with loss of preload reserve and contractile impairment secondary to LV dilation and eccentric hypertrophy [[18], [19], [20]]. However, in patients with BAV-AR and smaller AVAi, additional pressure overload promotes myocardial thickening and limits LV dilation from AR [21,22], conserving preload reserve and systolic function even without significant AS [23,24]. Our findings expand on previous observations [25], demonstrating that patients with BAV-AR had more concentric hypertrophy and more responsive Frank-Starling feedback, with milder decline in LVEF during LV dilation despite more severe AR than patients with TAV. These imply that unique haemodynamic patterns in BAV-AR may be responsible for more preserved LV systolic function, accounting for better symptomatic status despite more severe valvular dysfunction.

Despite more advanced valvular disease, patients with BAV-AR had lower surgical risk and fewer postoperative adverse events than TAV-AR counterparts, likely driven by their younger age. Existing literature comparing AR in different AV morphologies report superior real-life survival in patients with BAV [7], largely attributable to earlier disease onset. Likewise, our findings show that patients with BAV-AR enjoyed less postoperative adverse outcomes before age-matching, showing that age was likely the main driver behind better prognosis for BAV-AR. Nevertheless, the early onset of asymptomatic but severe valvular dysfunction in patients with BAV-AR with preserved systolic function demands improved risk stratification using age-independent predictors beyond LVEF and LVESD.

To this end, AVAi and LVMi emerge as key determinants of postoperative outcomes in patients with moderate and severe AR. Both AVA and LVMi are established predictors of mortality and adverse outcomes in patients with significant AR and BAV [15,26,27]. In patients with BAV-AR, reduced AVAi generates additional pressure overload causing myocardial thickening and fibrosis, preceding poorer outcomes without significant LVEF impairment and LV dilation [28]. Previous studies demonstrate that concomitant AR predicted poorer outcomes in patients with severe AS [29]. Concordantly, our findings show that AVAi and LVMi portend significantly poorer postoperative outcomes in patients with isolated significant AR, regardless of age and valvular morphology. The predictive value of AVAi and LVMi persisted after adjustment and age-matching, further demonstrating their unique utility in risk stratification for younger patients with BAV with milder symptoms and preserved systolic function.

### 4.1 Clinical implications

Current surgical recommendations for AR rely mainly on LVEF and LVESD [11], regardless of AV morphology [11]. However, the emergence of more sensitive prognosticators calls for a re-evaluation of current surgical thresholds [30]. This is especially true in BAV-AR, where LVEF and LVESD may overlook critical valvular and myocardial changes, better reflected by AVAi and LVMi for timely intervention of younger and less symptomatic patients with severe valvular disease.

Our study also clearly demonstrates that patients with BAV-AR harbor more aggressive yet silent valvular disease at a younger age, requiring closer monitoring and precise surgical timing before heart failure and symptomatic onset. Notably, previous studies show patients with BAV experience more postoperative HF readmissions, despite younger age and fewer comorbidities [24]. Accordingly, AVAi evaluation forms an important age-independent component of comprehensive preoperative assessment for patients with BAV-AR, along with important non-valvular comorbidities including aortic dilation and dissection [31].

### 4.2 Limitations

This study was a single-centre, retrospective study with inherent limitations. The sample size of BAV-AR was small but comparable to previous studies [24], and findings on LV function and adverse events may benefit from future studies. Echocardiographic assessment of the AV may be influenced by factors including hyperdynamic circulation, valvular calcification and heterogenous morphology in this cohort. The patterns and prognostic value of GLS, diastolic dysfunction and biomarkers in surgical AR patients await further investigation. The incremental prognostic value of AVAi and LVMi were modest but statistically significant, and would benefit from validation in future studies. The retrospective nature of this study also cannot establish causation between these parameters and postoperative outcomes, which would benefit from future prospective studies.

## 5 Conclusion

In patients requiring SAVR for isolated significant AR, patients with BAV had more advanced valvular pathology despite being a decade younger and less symptomatic, accompanied with more preserved systolic function during LV dilation. AVAi and LVMi were independently associated with postoperative adverse outcomes, and may inform close monitoring and early treatment among the significantly younger BAV-AR cohort.

## CRediT authorship contribution statement

**Ching-Yan Zhu:** Writing – review & editing, Writing – original draft, Visualization, Methodology, Investigation, Formal analysis, Data curation, Conceptualization. **Jing-Nan Zhang:** Methodology, Investigation, Formal analysis, Data curation, Conceptualization. **Ferit Böyük:** Methodology, Investigation, Formal analysis, Data curation, Conceptualization. **Yi-Kei Tse:** Methodology, Investigation, Formal analysis, Data curation, Conceptualization. **Yan Chen:** Methodology, Investigation, Formal analysis, Data curation, Conceptualization. **Mei-Zhen Wu:** Methodology, Investigation, Formal analysis, Data curation, Conceptualization. **Qing-Wen Ren:** Methodology, Investigation, Formal analysis, Data curation, Conceptualization. **Jia-Yi Huang:** Methodology, Investigation, Formal analysis, Data curation, Conceptualization. **Ran Guo:** Methodology, Investigation, Formal analysis, Data curation, Conceptualization. **Wen-Li Gu:** Methodology, Investigation, Formal analysis, Data curation, Conceptualization. **Daniel Tai-Leung Chan:** Resources, Project administration, Investigation, Data curation. **Frank A. Flachskampf:** Writing – review & editing, Supervision. **Kai-Hang Yiu:** Writing – review & editing, Writing – original draft, Supervision, Conceptualization.

## Source of funding

This study was supported by the Undergraduate Research Fellowship Programme, 10.13039/501100003803HKU, the 10.13039/501100001809National Natural Science Foundation of China (No. 82270400), the 10.13039/501100003453Natural Science Foundation of Guangdong Province (No. 2023A1515010731), and 10.13039/501100012151Sanming Project of Medicine in Shenzhen (No. SZSM202411021).

## Declaration of competing interest

The authors report no relationships that could be construed as a conflict of interest.

## Data Availability

The data that support the findings of this study are available from the corresponding author upon reasonable request.

## Appendix A Supplementary data

Supplementary material Image 1
