# Supplementary material for: Aortic valve replacement in patients with significant aortic regurgitation: Comparing bicuspid and tricuspid aortic valve outcomes
Source: Am Heart J Plus. 2025 Sep 12;59:100617. doi: 10.1016/j.ahjo.2025.100617 (PMC12465045; doi:10.1016/j.ahjo.2025.100617)

Supplementary Material

**Aortic Valve Replacement in Patients with Significant Aortic Regurgitation: Comparing Bicuspid and Tricuspid Aortic Valve Outcomes**

**Supplementary tables**

Supplementary table 1: Univariate Cox Regression Analysis for adverse events

Supplementary table 2: Baseline characteristics after exact age-matching

Supplementary table 3: Echocardiographic characteristics after exact age-matching

Supplementary table 4: Multivariate Cox regression analysis for adverse events after age-matching

Supplementary table 5: Indications for concomitant aortic surgery

Supplementary Table 6. AVAi in patients with BAV or TAV stratified by LVOT measurements

**Supplementary figures**

Figure S1. Kaplan-Meier analysis of survival and heart failure rehospitalization after SAVR according to AV phenotype in patients with significant AR.

Figure S2a: interaction between AV phenotype and indexed LV end-diastolic volume (LVEDVi) on indexed stroke volume (SVi) after age-matching. P for interaction = 0.007.

Figure S2b: interaction between AV phenotype and left ventricular end-systolic diameter (LVESD) on left ventricular ejection fraction (LVEF) after age-matching. P for interaction = 0.02.

Figure S3: Kaplan-Meier analysis of composite adverse outcomes after SAVR according to AV phenotype in patients with aortic regurgitation requiring surgery after age-matching.

Supplementary table 1: Univariate Cox Regression Analysis for adverse events

| Variable | HR (95% CI) | p-value |
| --- | --- | --- |
| **BAV** | **0.50 (1.07-3.80)** | **0.029** |
| **Age** | **1.04 (1.02-1.06)** | **<0.001** |
| Male sex | 1.08 (0.65-1.80) | 0.758 |
| Body mass index | 0.99 (0.94-1.05) | 0.836 |
| Body surface area | 0.38 (0.14-1.05) | 0.062 |
| **Diabetes** | **3.30 (1.79-6.08)** | **<0.001** |
| **Hypertension** | **2.19 (1.43-3.37)** | **<0.001** |
| Dyslipidemia | 1.56 (0.90-2.73) | 0.115 |
| Aortic aneurysm | 0.84 (0.53-1.35) | 0.480 |
| **Heart failure** | **5.75 (3.22-10.19)** | **<0.001** |
| **Creatinine** | **1.00 (1.00-1.00)** | **<0.001** |
| **Haemoglobin** | **0.80 (0.71-0.91)** | **<0.001** |
| Aspirin | 1.67 (0.90-3.10) | 0.104 |
| Statin | 0.95 (0.58-1.56) | 0.860 |
| Warfarin | 0.64 (0.32-1.30) | 0.215 |
| RAAS inhibitors | 2.69 (0.85-8.52) | 0.092 |
| Beta-blockers | 1.84 (0.89-3.82) | 0.101 |
| **Calcium channel blockers** | **2.09 (1.27-3.45)** | **0.004** |
| **Diuretics** | **4.38 (1.61-11.96)** | **0.004** |
| Total cross clamp time | 1.00 (1.00-1.01) | 0.356 |
| Total bypass time | 1.00 (1.00 – 1.01) | 0.310 |
| **EuroSCORE II** | **1.03 (1.01-1.05)** | **0.012** |
| **LVEF** | **0.97 (0.96-0.99)** | **0.006** |
| LVEDVi | 1.00 (1.00-1.00) | 0.526 |
| **LVESVi** | **1.01 (1.00-1.01)** | **0.014** |
| **GLS** | **1.09 (1.03-1.15)** | **0.002** |
| **Interventricular septum thickness** | **1.15 (1.07-1.25)** | **<0.001** |
| LVEDD | 1.00 (0.98-1.03) | 0.687 |
| LVESD | 1.02 (1.00-1.04) | 0.084 |
| LV posterior wall thickness | 1.09 (0.98-1.20) | 0.110 |
| **LV mass index** | **1.01 (1.00-1.01)** | **0.001** |
| Relative wall thickness | 1.02 (1.00-1.04) | 0.132 |
| AV maximal velocity | 1.08 (0.79-1.46) | 0.632 |
| AV maximal gradient | 1.00 (0.99-1.02) | 0.770 |
| AV mean gradient | 1.00 (0.97-1.03) | 0.958 |
| AR pressure half-time | 1.00 (1.00 – 1.00) | 0.831 |
| AR vena contracta width | 1.04 (0.97-1.12) | 0.285 |
| AR jet width | 1.01 (0.98-1.04) | 0.675 |
| AR regurgitant volume | 1.00 (0.99-1.01) | 0.556 |
| AR regurgitant fraction | 1.01 (0.99-1.03) | 0.452 |
| **AV area index** | **0.70 (0.50-0.99)** | **0.041** |
| Sinus of Valsalva diameter index | 1.00 (0.96-1.04) | 0.888 |
| Sino-tubular junction diameter index | 1.02 (0.98-1.06) | 0.348 |
| Ascending aorta diameter index | 1.04 (1.00-1.07) | 0.054 |
| **Left atrial volume index** | **1.00 (1.00-1.01)** | **0.001** |

Abbreviations: BAV, bicuspid aortic valve; LV, left ventricle; LVEF, left ventricular ejection fraction; LVEDVi, indexed left ventricular end-diastolic volume; LVESVi, indexed left ventricular end-systolic volume; GLS, left ventricular global longitudinal strain; LVEDD, left ventricular end-diastolic diameter; LVESD, left ventricular end-systolic diameter; AV, aortic valve; AR, aortic regurgitation.

Supplementary table 2: Baseline characteristics after exact age-matching

| **Variable** | **Overall,** N = 210 | **BAV**, N = 60 | **TAV**, N = 150 | **p-value** |
| --- | --- | --- | --- | --- |
| Demographic and physiological data | | | | |
| **Age, y** | **56 (11)** | **53 (10)** | **56 (11)** | **0.044** |
| Male sex | 159 (76%) | 50 (83%) | 109 (73%) | 0.103 |
| Body mass index, kg/m^2^ | 24.1 (3.8) | 24.1 (3.2) | 24.1 (4.0) | 0.908 |
| Body surface area, m^2^ | 1.74 (0.21) | 1.77 (0.19) | 1.72 (0.22) | 0.115 |
| Systolic blood pressure, mmHg | 132 (19) | 134 (22) | 132 (18) | 0.612 |
| Diastolic blood pressure, mmHg | 72 (13) | 74 (13) | 72 (13) | 0.438 |
| Clinical data | | | | |
| Diabetes | 9 (4.3%) | 0 (0%) | 9 (6.0%) | 0.063 |
| Hypertension | 38 (18%) | 12 (20%) | 26 (17%) | 0.650 |
| Dyslipidaemia | 24 (11%) | 9 (15%) | 15 (10%) | 0.304 |
| Aortic aneurysm | 67 (32%) | 21 (35%) | 46 (31%) | 0.543 |
| Heart failure | 101 (48%) | 25 (42%) | 76 (51%) | 0.238 |
| Coronary artery disease | 10 (30%) | 2 (14%) | 8 (42%) | 0.131 |
| Atrial fibrillation | 26 (12%) | 4 (6.7%) | 22 (15%) | 0.112 |
| Laboratory data | | | | |
| Creatinine, mmol/L | 104 (71) | 93 (33) | 109 (81) | 0.084 |
| Haemoglobin, g/dL | 12.95 (1.88) | 13.18 (1.50) | 12.85 (2.01) | 0.264 |
| Medications | | | | |
| Aspirin | 26 (17%) | 5 (11%) | 21 (19%) | 0.533 |
| Statin | 51 (32%) | 11 (24%) | 40 (36%) | 0.149 |
| Warfarin | 144 (92%) | 43 (93%) | 101 (91%) | 0.757 |
| RAAS inhibitors | 193 (92%) | 54 (92%) | 139 (93%) | 0.777 |
| Beta-blockers | 180 (86%) | 52 (88%) | 128 (85%) | 0.598 |
| Calcium channel blockers | 131 (63%) | 37 (63%) | 94 (63%) | 0.995 |
| Diuretics | 178 (85%) | 47 (80%) | 131 (87%) | 0.160 |
| Preoperative levosimendan | 6 (2.9%) | 2 (3.3%) | 4 (2.7%) | >0.999 |
| NYHA functional class | | | | 0.226 |
| 1 | 60 (29%) | 14 (23%) | 46 (31%) |  |
| 2 | 115 (55%) | 39 (65%) | 76 (51%) |  |
| 3 | 32 (15%) | 6 (10%) | 26 (17%) |  |
| 4 | 3 (1.4%) | 1 (1.7%) | 2 (1.3%) |  |
| Baseline valvular data | | | | |
| BAV type | | | | |
| 0 |  | 4 (6.8%) |  |  |
| 1 (LN) |  | 4 (6.8%) |  |  |
| 1 (LR) |  | 41 (69%) |  |  |
| 1 (RN) |  | 9 (15%) |  |  |
| 2 |  | 1 (1.7%) |  |  |
| AR grade | | | | 0.640 |
| Moderate | 48 (23%) | 15 (25%) | 33 (22%) |  |
| Severe | 162 (77%) | 45 (75%) | 117 (78%) |  |
| NYHA: New York Heart Association. AS: aortic stenosis. AR: aortic regurgitation. BAV: bicuspid aortic valve. | | | | |

Supplementary table 3: Echocardiographic characteristics after exact age-matching

| **Variable** | **Overall,** N = 210 | **BAV**, N = 60 | **TAV**, N = 150 | **p-value** |
| --- | --- | --- | --- | --- |
| Left ventricular ejection fraction, % | 51 (11) | 50 (11) | 51 (11) | 0.539 |
| LV end-diastolic volume index, mL/m^2^ | 135 (59) | 133 (54) | 136 (62) | 0.734 |
| LV end-systolic volume index, mL/m^2^ | 70 (41) | 68 (33) | 70 (44) | 0.609 |
| Stroke volume index, mL/m^2^ | 66 (27) | 66 (29) | 66 (26) | 0.995 |
| Interventricular septum thickness, mm | 12.67 (2.39) | 12.98 (1.94) | 12.54 (2.54) | 0.174 |
| Left ventricular end-diastolic diameter, mm | 60 (10) | 61 (11) | 59 (9) | 0.348 |
| Left ventricular end-systolic diameter, mm | 44 (11) | 45 (12) | 44 (10) | 0.636 |
| Left ventricular posterior wall thickness, mm | 11.77 (2.08) | 12.18 (2.13) | 11.60 (2.04) | 0.072 |
| Left ventricular mass index, g/m^2^ | 190 (60) | 198 (62) | 186 (59) | 0.196 |
| Relative wall thickness, % | 41 (10) | 42 (11) | 40 (10) | 0.290 |
| Left ventricular global longitudinal strain, % | -16.9 (4.7) | -17.4 (4.7) | -16.7 (4.8) | 0.465 |
| **AV maximal velocity, m/s** | **2.20 (0.72)** | 2.55 (0.86) | 2.06 (0.61) | <0.001 |
| **AV maximal gradient, mmHg** | **21 (15)** | 29 (20) | 18 (12) | <0.001 |
| **AV mean gradient, mmHg** | **11 (9)** | 16 (11) | 9 (6) | <0.001 |
| **AV area index, cm^2^/m^2^** | **1.73 (0.75)** | 1.57 (0.87) | 1.81 (0.69) | 0.064 |
| AR vena contracta width, mm | 6.58 (2.72) | 6.83 (3.03) | 6.48 (2.60) | 0.440 |
| **AR jet width, mm** | **19 (8)** | 17 (7) | 20 (8) | 0.010 |
| AR pressure half time, ms | 369 (190) | 390 (165) | 361 (199) | 0.298 |
| **AR regurgitant volume, ml** | **70 (30)** | 80 (33) | 66 (28) | 0.013 |
| AR regurgitant fraction, % | 61 (17) | 63 (20) | 61 (16) | 0.499 |
| Sinus of Valsalva diameter index, mm/m^2^ | 23.4 (5.2) | 23.0 (4.3) | 23.5 (5.5) | 0.432 |
| Sino-tubular junction diameter index, mm/m^2^ | 20.1 (5.1) | 20.0 (4.3) | 20.1 (5.4) | 0.858 |
| Ascending aorta diameter index, mm/m^2^ | 22.8 (5.3) | 23.6 (5.1) | 22.4 (5.3) | 0.152 |
| **Left atrial volume index, ml/m^2^** | **56 (33)** | 46 (19) | 60 (36) | <0.001 |
| E wave, m/s | 0.90 (0.36) | 0.85 (0.29) | 0.92 (0.38) | 0.183 |
| A wave, m/s | 0.68 (0.26) | 0.68 (0.26) | 0.68 (0.27) | 0.989 |
| E/A ratio | 1.48 (0.78) | 1.36 (0.59) | 1.53 (0.84) | 0.151 |
| Tricuspid regurgitation maximal velocity, m/s | 2.71 (0.48) | 2.72 (0.42) | 2.70 (0.50) | 0.880 |

Abbreviations: LV, left ventricle; AV, aortic valve; AR, aortic regurgitation

Supplementary table 4: Multivariate Cox regression analysis for adverse events after age-matching*

| Variable | HR (95% CI) | p-value |
| --- | --- | --- |
| Bicuspid aortic valve | 0.65 (0.32-1.32) | 0.236 |
| **LVMi** | **1.01 (1.00-1.01)** | **0.042** |
| **AVAi** | **0.57 (0.35-0.92)** | **0.022** |
| LAVi | 1.00 (1.00-1.01) | 0.056 |
| *adjusted for diabetes, hypertension, LVEF, LVESD | | |

Abbreviations: LVMi, indexed left ventricular mass; AVAi, indexed aortic valve area; LAVi, indexed left atrial volume; LVEF, left ventricular ejection fraction; LVESD, left ventricular end-systolic diameter

Supplementary table 5: Indications for concomitant aortic surgery

| **Surgical indication** | **Overall,** N = 41 | **BAV**, N = 9 | **TAV**, N = 32 |
| --- | --- | --- | --- |
| Aortic aneurysm | 24 (58%) | 6 (67%) | 18 (56%) |
| Aortic root dilation | 9 (22%) | 3 (33%) | 6 (19%) |
| Dissecting aortic aneurysm | 5 (12%) | - | 5 (16%) |
| Infective endocarditis | 3 (7.3%) | - | 3 (9%) |

Supplementary Table 6. AVAi in patients with BAV or TAV stratified by LVOT measurements

| Subgroup | Overall | BAV | TAV | p-value |
| --- | --- | --- | --- | --- |
| LVOT VTI < 22cm | 1.38 (0.46) (n=89) | 1.17 (0.38) (n=27) | 1.46 (0.46) (n=62) | 0.003 |
| LVOT diameter <= 20mm | 1.39 (0.51) (n=62) | 1.01 (0.30) (n=8) | 1.45 (0.52) (n=54) | 0.004 |
| LVOT diameter > 20mm | 1.79 (0.73) (n=251) | 1.57 (0.86) (n=62) | 1.87 (0.66) (n=189) | 0.016 |

Figure S1. Kaplan-Meier analysis of survival and heart failure rehospitalization after SAVR according to AV phenotype in patients with significant AR.

Survival


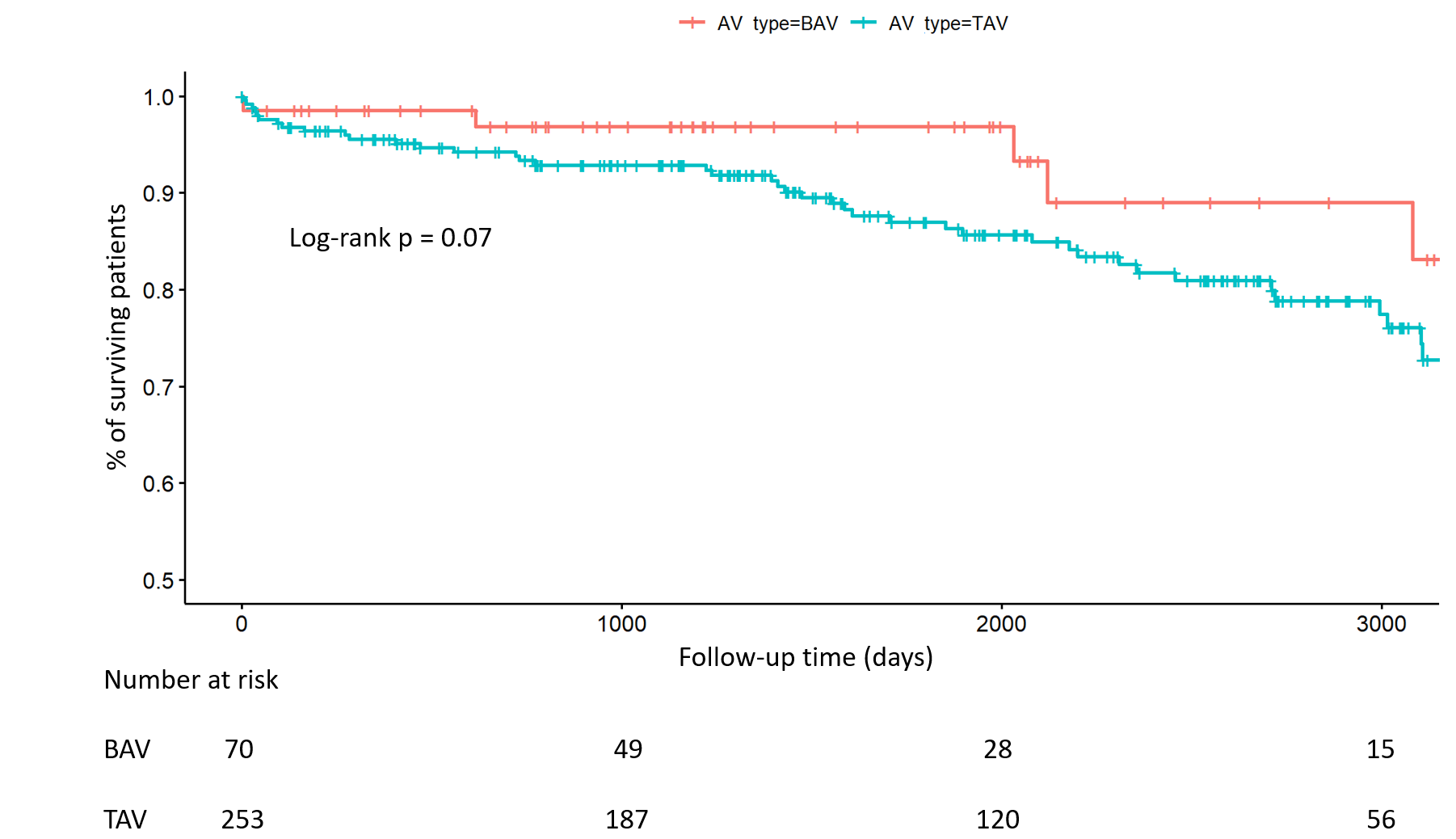


Heart failure rehospitalization (HFR)


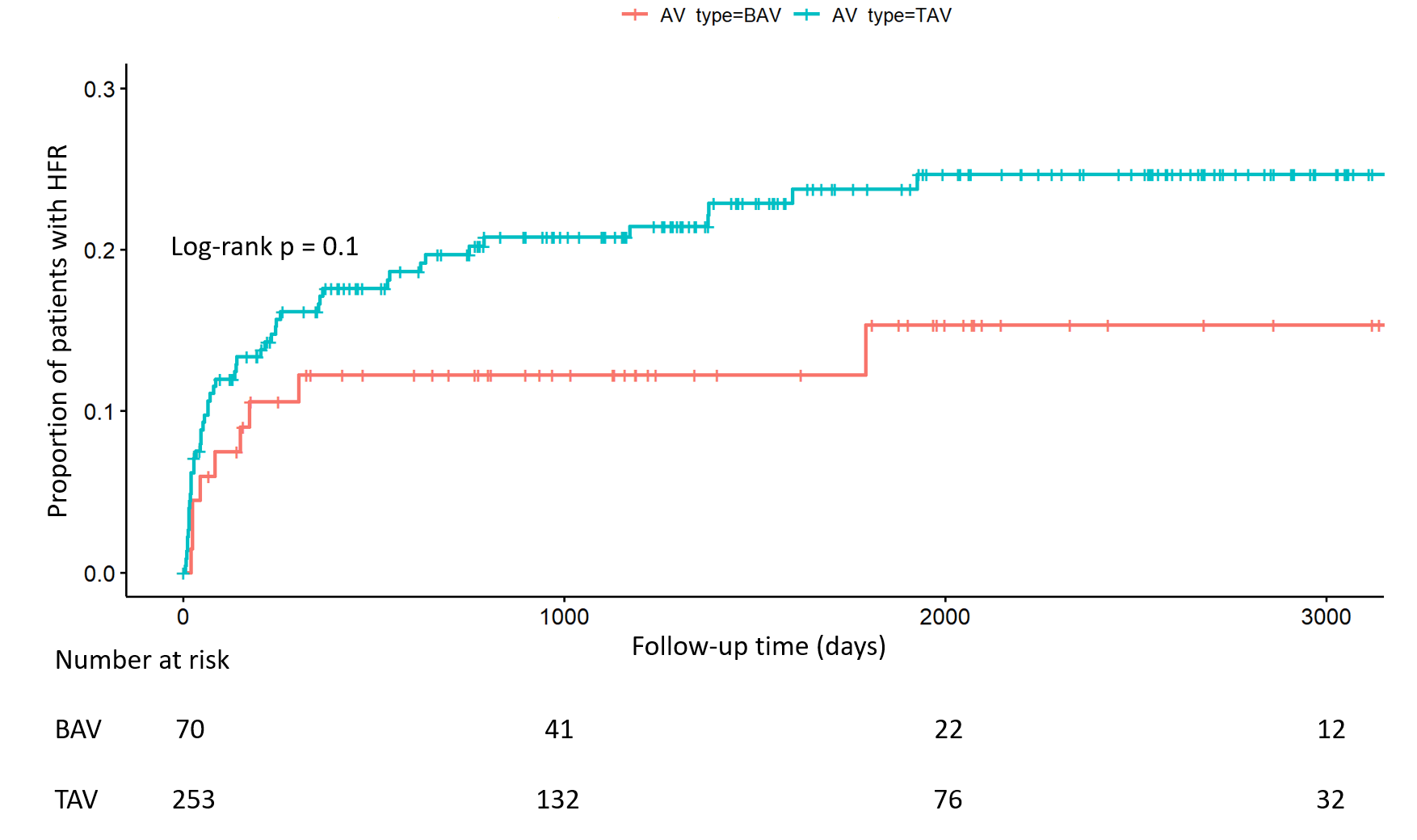


Figure S2a: interaction between AV phenotype and indexed LV end-diastolic volume (LVEDVi) on indexed stroke volume (SVi) after age-matching. P for interaction = 0.007.


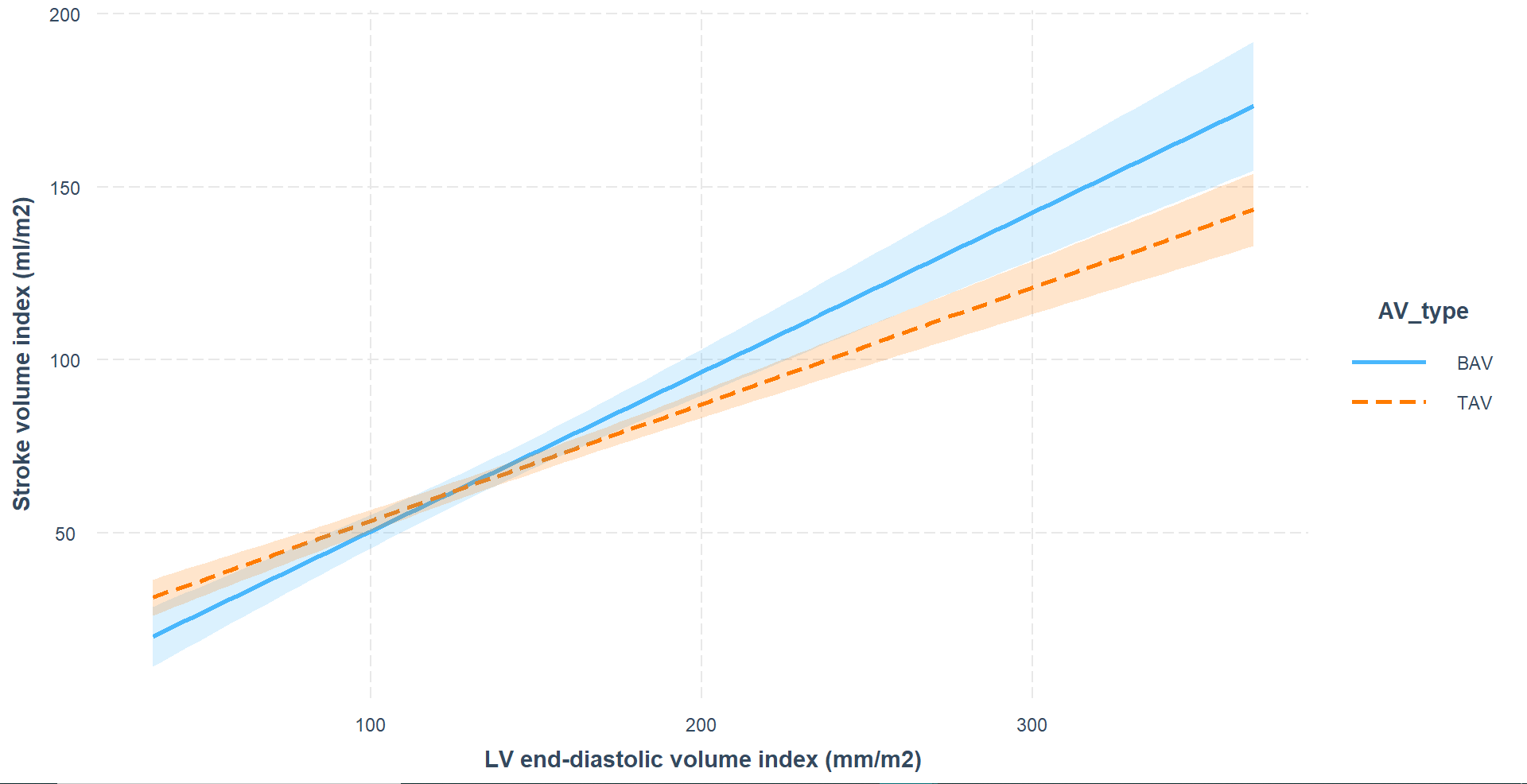


Figure S2b: interaction between AV phenotype and left ventricular end-systolic diameter (LVESD) on left ventricular ejection fraction (LVEF) after age-matching. P for interaction = 0.02.


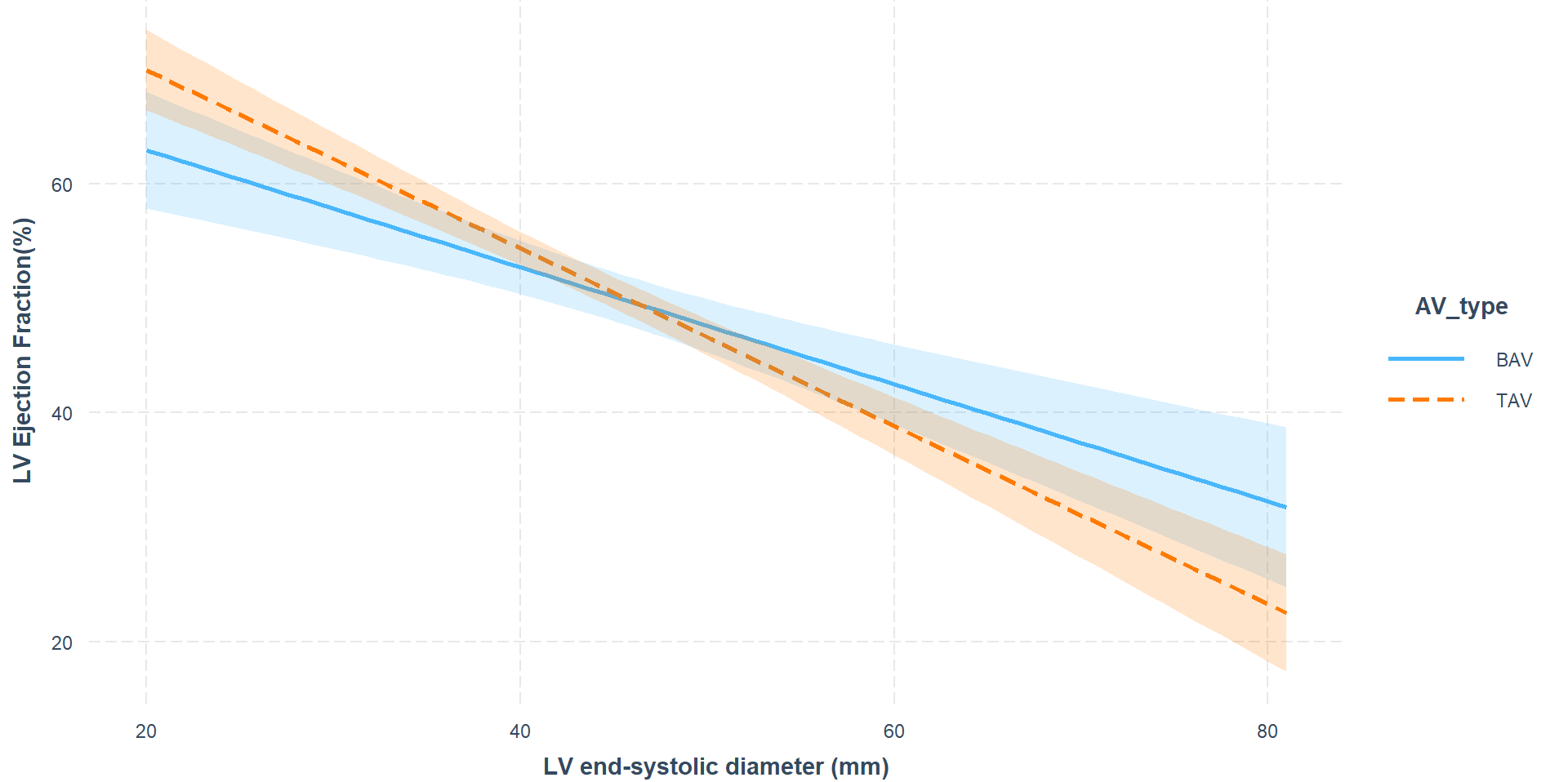


Figure S3: Kaplan-Meier analysis of composite adverse outcomes after SAVR according to AV phenotype in patients with aortic regurgitation requiring surgery after age-matching.


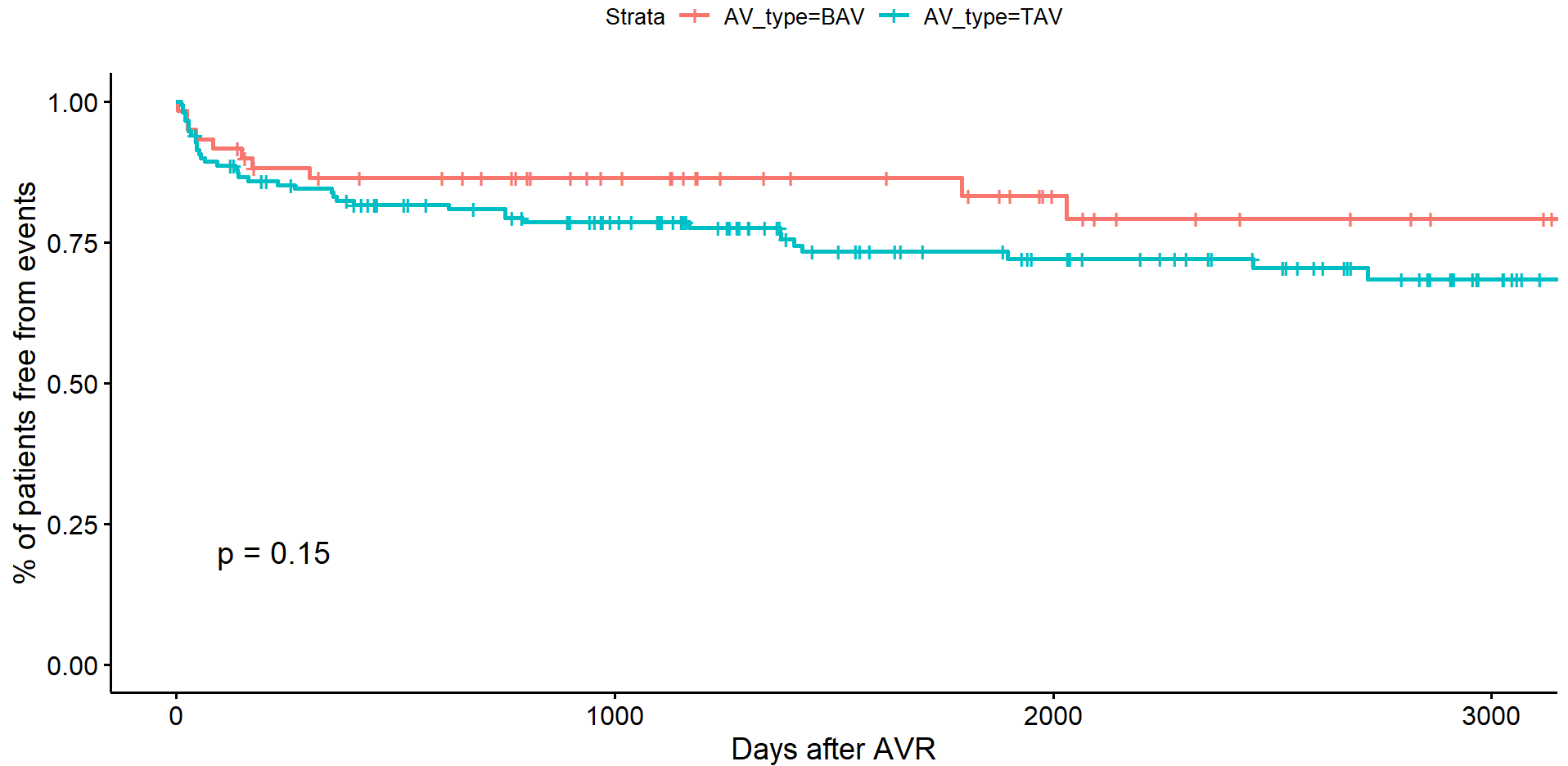

Supplement: Supplementary file 1 — Supplementary material [file mmc1.docx]
